# High performance mode (de)multiplexer assisted with a microring resonator on the lithium niobate-on-insulator platform

**DOI:** 10.1515/nanoph-2025-0146

**Published:** 2025-07-07

**Authors:** Wenbing Jiang, Jiang Qu, Yu Guo, Boyu Zhang, Jia Du, Xiongping Bao, Xiao Chen, Weibiao Chen, Libing Zhou

**Affiliations:** Wangzhijiang Innovation Center for Laser, Aerospace Laser Technology and System Department, Shanghai Institute of Optics and Fine Mechanics, Chinese Academy of Sciences, Shanghai, 201800, China; Center of Materials Science and Optoelectronics Engineering, University of Chinese Academy of Sciences, Beijing, 100049, China; School of Advanced Interdisciplinary Sciences, University of Chinese Academy of Sciences, Beijing, 100049, China; Department of Optics and Optical Engineering, University of Science and Technology of China, Hefei, 230026, China

**Keywords:** asymmetric directional coupler, thin film lithium niobate, mode (de)multiplexer, integrated acousto-optic modulators, microring resonator

## Abstract

The high extinction ratio mode (de)multiplexer is a pivotal component in high capacity mode-division multiplexing data communication and nascent on-chip intermodal acousto-optic modulators. Up to now, high performance on-chip mode (de)multiplexers are still lacking for integrated AOMs on the lithium niobate-on-insulator platform. In this paper, we propose and demonstrate an innovative scheme to achieve high extinction ratio signal routing for acousto-optic modulation, by leveraging a two-mode (de)multiplexer in conjunction with a high-*Q* racetrack microring resonator. The integrated devices are fabricated with one-step electron beam lithography and dry etching processes. The demonstrated two-mode (de)multiplexer boasts the excellent intermodal crosstalk below −20 dB and the on-chip insertion loss of less than 1.92 dB within the wavelength range of 1,514–1,580 nm. With the reinforcement of the microring resonator filter, the carrier signal can be suppressed thoroughly and the measured extinction ratio attains over 30 dB. Our proof-of-principle investigations have provided a feasible and compact solution to implement practical intermodal AOMs in LNOI for photonic and quantum information process, microwave photonics, and LiDAR.

## 1 Introduction

The lithium niobate-on-insulator (LNOI) platform serves as a promising candidate for the next generation photonic integrated circuits (PIC) for optical data communication, sensing, and computation by virtue of the versatile material properties combining large electro-optic, photoelastic, and nonlinear-optic effects, along with a wide transparency window [1], [2], [3]. In recent years, thanks to the breakthrough of wafer-scale LNOI manufacturing and low-loss nanofabrication of LNOI PIC, unprecedented device performance and chiplet functionalities have been achieved on the basis of the tightly confined optical mode in LNOI waveguides, e.g. high-speed electro-optic modulators (EOM) [4], [5], [6], compact acousto-optic modulators (AOM) [7], [8], [9], [10], [11], [12], [13], efficient frequency converters [14], [15], [16], and broadband optical frequency comb generators [17], [18], [19], [20]. Moreover, light sources [21], [22], [23], photodetectors [24], [25], waveguide amplifiers [26], [27], [28], and quantum photonic applications [29] have also been achieved on the LNOI platform.

Among the emerging integrated photonic devices in thin-film lithium niobate, AOMs have sustainedly attracted immense interest due to the extensive applications in photonic signal processing [30], [31], coherent quantum transduction [8], [32], and microwave photonics [11], [33], etc. Currently, most of reported on-chip AOMs mainly focus on the intramodal phase modulation, as demanded in microwave-to-optical conversion. Indeed, integrated single-sideband amplitude AOMs are urgently desired in many application scenarios, offering the solution of compact, low-power consumption, and multifunctional integration instead of the commercial bulk counterparts. So far, there are two mainstream approaches to realize integrated single-sideband AOMs, namely acoustic Bragg scattering [34], [35] and mode demultiplexing in the wake of intermodal modulation [30], [33]. Acoustic Bragg scattering can separate the modulated light from the incident light in the spatial domain, but at the expense of large chip footprints. For the second approach, the mode demultiplexer plays an indispensable role to individually route the modulated signal, because the optical mode parity undergoing the acousto-optic process has been changed indeed.

The mode (de)multiplexer has already been adopted in intermodal AOMs in the silicon photonics and thin-film AlScN platforms [36], [37], [38], [39], [40], [41]. However, the high extinction ratio (ER) mode demultiplexer on the LNOI platform for on-chip AOMs is still missing to date. As a matter of fact, mode (de)multiplexers in thin-film lithium niobate have been experimentally demonstrated for mode-division multiplexing (MDM) data communication. For example, four-channel TM mode (de)multiplexers exploiting photonic bound states in the continuum (BICs) were reported on an etchless LNOI platform [42]. An electro-optic reconfigurable two-mode (de)multiplexer was demonstrated at the C-band, exhibiting the crosstalk less than −16.9 dB [43]. *Y. Liu* et al. presented a four-mode (de)multiplexer in the silicon rich nitride loaded LNOI platform, using the tapered asymmetric directional coupler (ADC) structure [44]. All channels show the small insertion loss and low crosstalk below −16 dB over a broad bandwidth of 100 nm. Similar device configurations with stoichiometric Si_3_N_4_ loading waveguides on the LNOI platform have been adopted [45]. More recently, with dry-etching of LN films, mode (de)multiplexers based on LN ridge/stripe waveguides were demonstrated as well. For example, in Ref. [46], an impressive four-channel mode (de)multiplexer with low insertion loss less than 0.2 dB and crosstalk less than −19 dB has been experimentally achieved. The broadband (de)multiplexer based on LNOI stripe waveguide configurations, alleviating precise control of the etching depth, also exhibits the crosstalk below −10 dB for all the channels but suffers from large excess loss [47]. Although the performance of present mode (de)multiplexers is good enough for MDM high-capacity optical communication [48], the intermodal crosstalk doesn’t yet meet the requirements of on-chip AOMs or multimode photonics, generally exceeding 45 dB in analogy to bulk AOMs [49].

In this paper, we propose and experimentally verify a high ER two-mode (de)multiplexer assisted with a multimode racetrack microring resonator (MRR) on the LNOI platform. The device fabrication only needs one-step electron beam lithography (EBL) and dry etching process. Firstly, we optimize the constituent two-mode (de)multiplexer which presents the small on-chip insertion loss less than 1.92 dB and intermodal crosstalk below −20 dB at the wavelength ranging from 1,514 to 1,580 nm. Subsequently, harnessing the elaborated design of multimode waveguides and critical coupling conditions for the MRR, the measured full width at half maximum (FWHM) of MRR resonances at the C-band are 13.5 and 6.6 pm, respectively, satisfying the need of high-resolution filtering of the proposed scheme. Eventually, the MRR-assisted two-mode demultiplexer experimentally demonstrates a high ER exceeding 30 dB, attaining the maximum value of 35 dB.

## 2 Principle and device design

**Figure 1:**
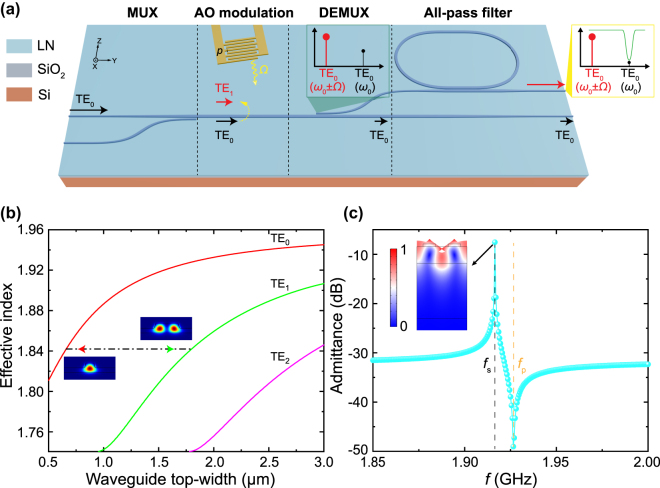
Mockup of the device configuration and simulations of photonics and acoustics. (a) Schematic illustration of the high performance mode (de)multiplexer assisted with a microring filter on the lithium niobate-on-insulator platform. MUX: multiplexer; AO modulation: acousto-optic modulation; DEMUX: demultiplexer. *ω*_0_: input optical frequency; Ω: acoustic frequency. The schematic spectra before and after the MRR are denoted in the inset, and the crystallographic coordinate system is indicated as well. To demonstrate high-ER performance in actual devices, the AO modulation stage is omitted, with its functionality instead emulated by the MUX stage. (b) Calculated effective indices of the three lowest-order TE modes as a function of the waveguide top-width. The dash line marks the waveguide widths satisfying the phase matching condition, where the simulated E-field profiles of TE_0_ and TE_1_ modes are shown simultaneously. (c) The simulated admittance of the surface acoustic wave circuit. The inset shows the acoustic mode profile at the SAW resonant frequency *f*_s_ = Ω/2*π*, while *f*_p_ denotes the SAW anti-resonant frequency.

The schematic diagram of the proposed high-ER mode (de)multiplexer for the AOM is illustrated in Figure 1a. We start with a two-channel mode (de)multiplexer based on the ADC structure to realize the modal separation between the modulated TE_1_ mode and the injected TE_0_ mode [50], [51], [52], [53], [54], [55]. Here, the mode multiplexing stage emulates the mode conversion from TE_0_ to TE_1_ mode in the bus waveguide, analogous to the intermodal AO modulation. As the surface acoustic wave (SAW) with a non-zero axial wavevector and acoustic frequency Ω is actuated by the tilted interdigital transducer (IDT), the injected TE_0_ mode (frequency: *ω*_0_) will be scattered into the modulated TE_1_ mode (frequency: *ω*_0_ ±Ω) under the anti-Stokes or Stokes scattering process. Subsequently, the modulated TE_1_ mode will transfer into TE_0_ mode at the cross port with the same frequency, while a tiny amount of the injected TE_0_ mode leaks into the cross port, which thus affects the modulation ER. Due to the frequency difference between these two TE_0_ modes, it’s feasible to co-integrate the all-pass filter based on an MRR to filter out the leaked TE_0_ mode, anticipating to enhance the modulation ER. The schematic spectra of the modulation and carrier signals before (after) the MRR are compared in Figure 1a, when the resonance frequency of MRR is aligned to *ω*_0_ and the modulation signal locates the pass band simultaneously. However it is not trivial to achieve the high-resolution and effective filtering of leaked TE_0_ mode, because the modulation and probe tones are very close in the frequency domain, generally spanning from MHz to GHz levels [2]. Therefore, the elaborated design of the high *Q*-factor MRR and the low-crosstalk ADC should be executed.

Generally, we classify the device design into two parts: the mode (de)multiplexer and the critical coupling MRR. The proposed device is constructed on the X-cut LNOI platform where the thicknesses of thin-film lithium niobate and the buried oxide layer are 600 nm and 2 μm, respectively, and the silicon handling layer is 525 μm thick. To achieve two-mode (de)multiplexing based on the ADC, the phase-matching condition between the bus and access waveguides need to be rigidly satisfied. We simulated the waveguide eigenmode behaviors with varying waveguide widths as shown in Figure 1b, with optical propagation along the crystallographic Y-direction. In our case, the LNOI waveguide is half-etched to facilitate the efficient acousto-optic modulation, so the rib height is 300 nm. The sidewall etching angle is set as 72° according to our real fabrication process. To avoid the higher-order mode excitation, we choose the width of the bus waveguide being 1.8 μm. According to the phase-matching condition, TE_1_ mode in the bus waveguide converts to TE_0_ mode in the narrower access waveguide with the top-width of 0.66 μm, also at the single-mode condition. Additionally, we estimated the requirement of narrow-band filtering by simulating the acoustic frequency. The SAW wavelength is supposed to equal the width of the bus waveguide, to enable the maximal acousto-optic modulation efficiency [33], [36]. We adopted the piezoelectric simulation in the COMSOL Multiphysics software to extract the acoustic frequency for the SAW wavelength of 1.8 μm. The simulated admittance of the acoustic circuit is presented in Figure 1c, where a peak at *f*_s_ = 1.917 GHz in admittance marks the SAW resonant frequency. According to the calculation, the FWHM in the assisted MRR should be at least narrower than 15.3 pm at the C-band. Based on the phase-matching condition for the anti-Stokes process, the pitch *p* and the tilt angle *θ* of IDTs are expressed as:

(1) $$p=\frac{w\cos\theta }{2},$$

(2) $$\theta =\arctan\left[\frac{w}{\lambda \text{p}}\left(n_{\text{eff}1}-n_{\text{eff}2}\right)\right].$$

In Eqs. (1) and (2), *w* and *λ*_p_ denote the width of the bus waveguide in the ADC and the optical wavelength, respectively. *n*_eff1_ and *n*_eff2_ represent the effective refractive indices of guided TE_0_ and TE_1_ modes at *λ*_p_, respectively. The calculated parameters for the IDT are *p* = 0.894 μm and *θ* = 6.314° for the designed ADC. The calculated electromechanical coupling factor ${k_{\text{t}}}^{2}$ is 1.29 % according to the formula ${k_{\text{t}}}^{2}$ = $\pi^{2}/8\cdot \left({f_{\text{p}}}^{2}-{f_{\text{s}}}^{2}\right)/{f_{\text{s}}}^{2}$ [56].

**Figure 2:**
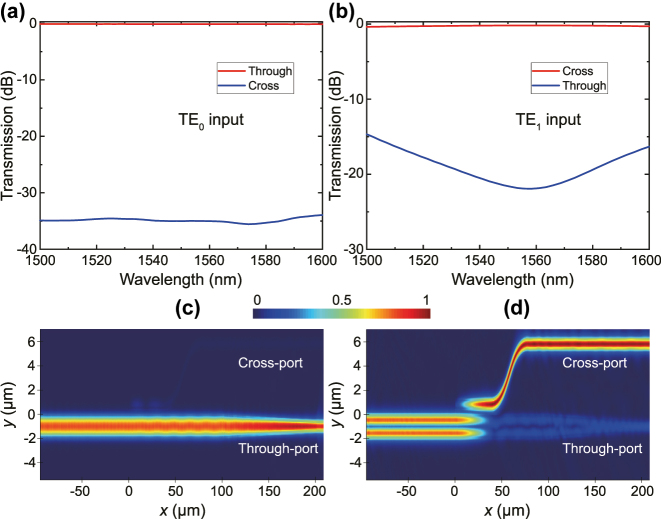
Simulated transmission spectrum of the ADC for the TE_0_ input (a) and the TE_1_ input (b). The simulated light propagation at the wavelength of 1,550 nm for TE_0_ (c) and TE_1_ (d) inputs, respectively.

The ER of the proposed device depends on the intermodal crosstalk of the mode (de)multiplexer, as well as the on-resonance ER and the FWHM bandwidth of the MRR simultaneously. Next, we simulated the intermodal crosstalk of the ADC with the 3D finite-difference time-domain (FDTD) solver (Lumerical). Figure 2 shows the simulated spectral transmission of the designed ADC and the corresponding light propagation under inputs of TE_0_ and TE_1_ modes, respectively. Initially, we optimize the coupling length *L*_*c*_ to maximize the coupling efficiency from TE_1_ mode in the bus waveguide to TE_0_ mode in the access waveguide. To ease the fabrication, the coupling gap is chosen to be 600 nm. The optimized mode coupling efficiency reaches over 96 % at 1,550 nm with *L*_*c*_ = 38 μm. In contrast, the injected TE_0_ mode maintains its propagation along the bus waveguide and hardly leaks into the cross-port, as seen in Figure 2c, due to the vast difference of effective refractive indices between the bus waveguide and the access waveguide. Accordingly, the input TE_1_ mode almost fully converts into the adjacent access waveguide because the phase-matching condition is met in Figure 2d. The residual TE_1_ mode emerges into a single-mode waveguide adiabatically through a linear-taper waveguide. The calculated transmission spectra for both inputs are displayed in Figure 2a and b, respectively. As seen in the simulation results, the excess losses for TE_0_ and TE_1_ modes are 0.12 dB ( < 0.14 dB) and 0.19 dB ( < 0.37 dB) at 1,550 nm (the wavelength range from 1,500 to 1,600 nm), respectively. The intermodal crosstalk levels for TE_0_ and TE_1_ modes are −34.9 dB ( < −33.8 dB) and −21.4 dB ( < −14.3 dB) at 1,550 nm (the wavelength range from 1,500 to 1,600 nm), respectively.

**Figure 3:**
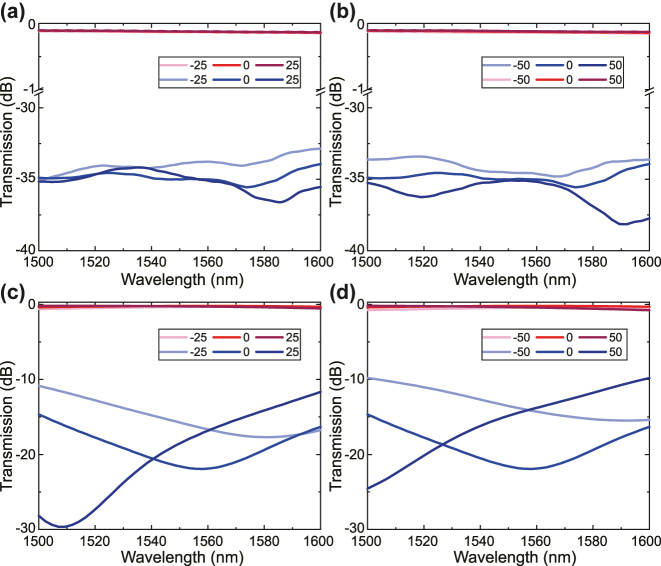
Simulated transmission spectrum of the ADC with the fabrication errors of the waveguide width at ± 25 nm (a) and ± 50 nm (b) for the TE_0_ input, and ± 25 nm (c) and ± 50 nm (d) for the TE_1_ input, respectively.

We further analyzed the fabrication tolerance of the ADC, as shown in Figure 3. The deviations of the waveguide width are assumed to be ±25 nm and ±50 nm in the analyses. As seen in Figure 3a and b, the intermodal crosstalk of TE_0_ mode seems robust even when the waveguide width deviates at ±50 nm. The intermodal crosstalk of TE_0_ mode remains less than −32 dB in the whole wavelength range. However, the through-port transmission of TE_1_ mode has a moderate sensitivity to the deviation of waveguide widths. As the waveguide width expands, there is a blue shift in the through-port transmission but the intermodal crosstalk is still less than −10 dB in the investigated wavelength range.

**Figure 4:**
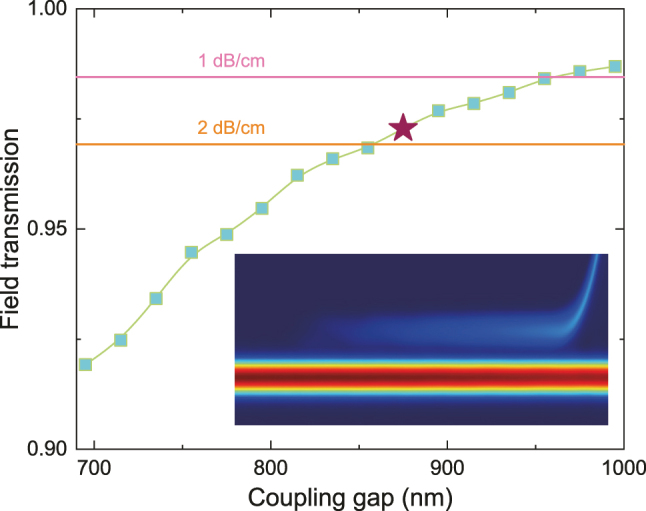
Design of the critical coupling MRR resonator. The main panel plots the field transmission coefficient *t* as a function of the coupling gap between the bus waveguide and the racetrack waveguide. The solid lines represent the corresponding values of *t*, satisfying the critical coupling condition, in the case of the waveguide propagation loss of 1 dB/cm and 2 dB/cm, respectively. The brown pentacle symbol denotes our designed gap, while the relevant simulated light propagation at 1,550 nm is displayed in the inset.

To abundantly filter out the residual input TE_0_ signal and let the modulated signal pass, a high *Q*-factor MRR with the critical coupling condition, which has a large ER at the resonance wavelength, is employed. Herein, a multimode waveguide coupling strategy is adopted to decrease the scattering loss from waveguide sidewall roughness [57], [58], improving the *Q*-factor and the filter solution. The coupling between the bus waveguide and the MRR is achieved with a straight direction coupler (DC) where the widths of the bus and racetrack waveguides are simultaneously set to 2 μm. To assure the dense free-spectral-range (FSR) to enhance ERs at as many wavelengths as possible, reduce the wavelength tuning range, and keep the device compact, we utilize the Euler bend to connect straight waveguides, as can obviate the intermodal crosstalk and bending loss as well [59]. We simulated the field transmission coefficient *t* as the coupling gap varies in Figure 4, where the coupling length is fixed as 100 μm. With the coupling gap increasing, *t* gradually increases to unity, entering under-coupled regimes. When *t* equals *a* = exp (-*α* *L*/2) which is the optical field attenuation per round trip *L* with the attenuation constant *α* [*m* ^−1^] [60], [61], the MRR operates at the expected critical coupling condition. We calculated the corresponding values of *t* for the estimated waveguide propagation loss of 1 dB/cm and 2 dB/cm respectively, as marked by the solid horizontal lines in Figure 4, deriving the respective gap sizes of 955 nm and 855 nm for the critical coupling condition of the MRR. In our experiments, we select the gap size as 875 nm taking into consideration the fabrication deviations and the LNOI waveguide propagation loss in our fabrication platform. The top view of light propagation in the DC coupling region is displayed in the inset, at the calculated power coupling efficiency of 5.36 %.

## 3 Device fabrication and measurement

**Figure 5:**
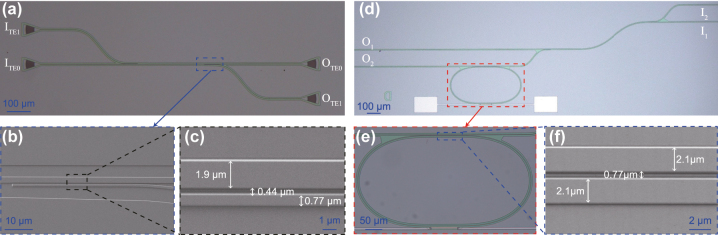
Optical microscope images and SEM images of the fabricated devices. (a) Optical microscope image of the mode (de)multiplexer. (b) and (c) Magnified SEM images of the ADC region. (d) Optical microscope image of the MRR-assisted mode (de)multiplexer. (e) Enlarged view of the integrated MRR. (f) SEM images of the coupling region in the MRR. The actual dimensions of the bus waveguide (upper), the coupling gap, and the waveguide in the MRR (lower) are indicated.

We fabricated the device on a commercial X-cut LNOI wafer (NANOLN) with the one-step electron beam lithography (EBL) and argon ions inductively coupled plasma etching (ICP) processes. The fabricated device is air cladding to enable future co-integration with SAW circuits. Figure 5a shows the optical microscope image of a fabricated mode (de)multiplexer with two inputs and two outputs. Figure 5b and c show the magnified scanning electron microscope (SEM) images and the measured dimensions of the ADC region, respectively. The grating couplers with the period of 1 μm and the filling factor of 0.68 are adopted to characterize the mode (de)multiplexer device. The overall structure of the MRR-assisted mode (de)multiplexer consists of the above-mentioned mode (de)multiplexer, a thermal-tuning MRR, and the edge couplers guiding light into and out of the chip, as displayed in Figure 5d. Herein we note that the edge coupler is applied, because of its smaller coupling loss, to assess the device performance of the MRR-assisted mode (de)multiplexer. Figure 5e shows the zoomed-in view of the coupling region in the MRR, where the actual dimensions of coupling waveguides and the coupling gap are indicated in Figure 5f, coincident with the design values in the case of fabrication errors. Furthermore, the actual etching depth and the etching angle are measured to about 290 nm and 72.7°, respectively, both close to the design values.

**Figure 6:**
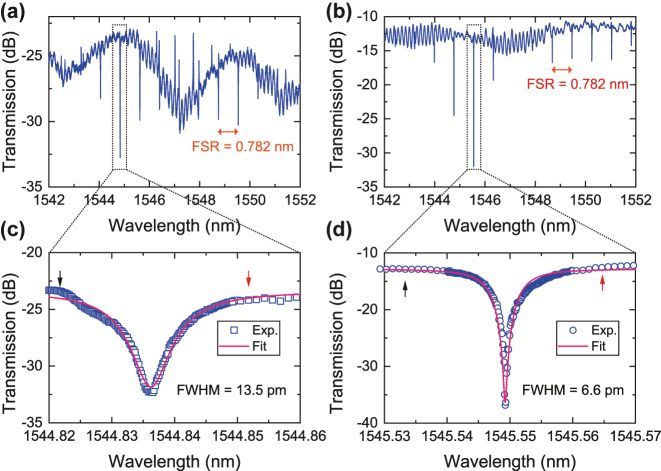
Full spectral response of device 1 (a) and device 2 (b). (c) and (d) The magnified experimental data and Lorenz fitting at the resonance. The locations of two sideband modulation signals are marked by the arrows.

Although the stand-alone mode (de)multiplexer demonstrates the small on-chip insertion loss and low crosstalk below −20 dB (see Supplementary Information S1), integrated AOMs adopting the mode demultiplexer still confront the issue of carrier leakage. In the following, we focus on characterizing the MRR-assisted mode (de)multiplexer to verify our design. The coupling loss of the edge coupler adopted for subsequent measurements is measured to be 7 dB/facet at 1,550 nm. Firstly, we selected two devices, labelled as device 1 and device 2, to assess the performance of integrated MRRs based on I_1_–O_2_ measurement ports. Figure 6a and b show the normalized full spectrum of MRRs for two devices at the wavelength resolution of 1.3 pm with an optical vector analyzer (Luna, OVA5100). The measured FSR is 0.782 nm, consistent with the simulation. For accurately estimating the internal loss and calibrating the resonance wavelength in the following ER measurement, we chose two prominent resonances at 1,544.836 nm and 1,545.549 nm to finely scan the detailed spectral response using our home-built measurement setup. The experimental data and Lorentz fitting are shown in Figure 6c and d, respectively. According to the fit, the extracted FWHM for both resonances are respective 13.5 pm and 6.6 pm, enough narrow to filter the carrier signal while allowing the modulated signal to pass. The two sideband tones with the detuning of 15.3 pm are located in the pass band, as indicated by the black and red arrows. The on-resonance ERs for two devices are 9 dB and 24 dB, respectively, indicating the potential for substantially improving the ER of stand-alone mode (de)multiplexers. The fitted intrinsic (coupling) quality factors *Q*_i_’s (*Q*_c_’s) for two devices are 3.54 (1.68) × 10^5^ and 5.01 (4.46) × 10^5^, respectively. Hence, the corresponding propagation losses of LN multimode waveguides are calculated to be 1.12 dB/cm and 0.79 dB/cm, respectively. The small transmitted power at the resonance wavelength and the nearly identical values of extracted *Q*_i_ and *Q*_c_ also demonstrate the critical-coupling condition of our designed MRRs.

**Figure 7:**
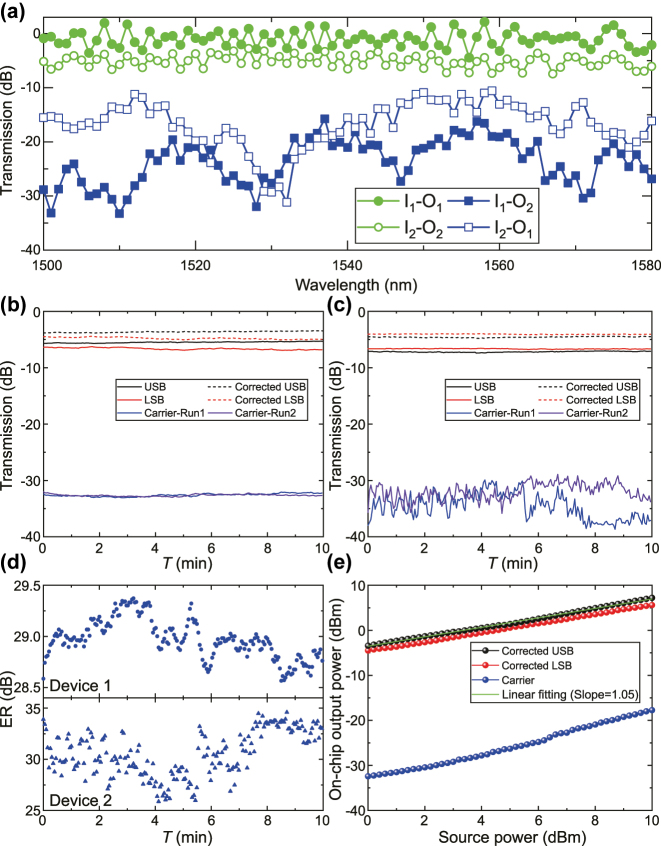
Measurement results of the integrated device. (a) Characterization of the LNOI mode (de)multiplexer in device 1. Monitoring spectra of USB, LSB, and carrier signals for device 1 (b), and device 2 (c). The corrected USB and LSB are both shown as well. (d) The extracted ERs at the first run measurement for device 1 and device 2. (e) On-chip output power versus incident laser power for device 1, along with a linear power fit applied to the corrected USB signal.

After accomplishing the calibration of stand-alone mode (de)multiplexers and the MRR, we characterized the ultimate ER of the MRR-assisted mode (de)multiplexer. Figure 7a displays the normalized transmission spectrum of the (de)multiplexer part in device 1, where the intermodal crosstalk for input TE_0_ mode is −19 dB at 1,550 nm, consistent with the measured results of the individual mode (de)multiplexer (see Supplementary Information Figure S1). The intermodal crosstalk of TE_0_ mode in device 2, degrading to about −10 dB resulting from larger fabrication deviations, is not shown here. We note that the oscillation of measured signals is due to the Fresnel reflection of the input and output chip facets [62].

As shown in Figure 7a, the average insertion losses of the integrated device are 1.0 dB and 5.1 dB for TE_0_ and TE_1_ modes, respectively. Compared with the individual mode (de)multiplexer on the same chip, the insertion loss of TE_1_ mode exhibits an increase of 1.5 dB, which is attributed to the additional on-chip insertion loss of the MRR (∼ 1.4 dB) (see Supplementary Information S2). For the integrated device applications to AOMs, where input signals are fed through the bus waveguide, only the mode demultiplexer integrated with the MRR is utilized to route modulation signals. Thus, by deducting the TE_0_-TE_1_ conversion loss of the mode multiplexer from the measured insertion loss of TE_1_ mode, we estimate the total insertion loss of the integrated device to be 3.25 dB. We point out that the TE_0_-TE_1_ conversion loss of the integrated device is somehow higher than that of the mode (de)multiplexer in Figure S1, because of the fabrication error which breaks the phase-matching condition of the ADC (see Supplementary Information S2). Since the modulation ER is determined by the difference between the total insertion loss of the integrated device and the crosstalk of TE_0_ mode, higher insertion loss induced by higher TE_0_-TE_1_ conversion loss and the MRR insertion loss will directly degrade the ER. Therefore, utilizing the adiabatic ADC with large fabrication tolerance and reducing the on-chip insertion loss of the MRR will be anticipated to mitigate the device insertion loss and hence improve the ER in future studies.

Based on the measurement results in Figure 6c and d, we fix the input carrier signal exactly at 1,544.836 nm and 1,545.549 nm from the I_1_ port, and monitor the transmission of the O_2_ port to assess the performance of carrier suppression. While resembling the actual modulation signals, we utilize the I_2_–O_2_ ports with the input signals at the detuning wavelength from the resonance by 15.3 pm, and monitor the transmission out of the O_2_ port to quantify the eventual ER. We repeat multiple segments of continuous measurements to confirm the reliability, each measurement lasting for 10 min, as seen in Figure 7b and c. To compensate for the TE_0_-TE_1_ mode conversion loss at the mode multiplexing stage, herein we correct the upper-sideband (USB) and lower-sideband (LSB) signals represented by dashed lines in Figure 7b and c. We numerate the ERs for two devices in Figure 7d. The extracted ER of device 1 is distinctly larger than 28.6 dB. For device 2, due to the perfect critical coupling condition of the MRR, there are evident variations in the transmission of carrier signals possibly caused by the thermal drift of the MRR resonance. Therefore, we average the measured ER of device 2, obtaining the mean ER over 30 dB and the maximal ER of 35 dB.

## 4 Discussion

Here we note that the measured ERs are based on the condition where the power of modulation signals equals that of probe signals, corresponding to the modulation efficiency of 50 %. In actual intermodal AOMs, the resulting ER is directly dependent on modulation efficiency. As shown in Figure 7e, the modulation signals exhibit a linear power response, indicating that the estimated ER of our device can reach up to ∼ 50 dB if the modulation efficiency approaches unity. Conversely, if the modulation efficiency drops to a few percent, the ER will degrade to a range of about 10–20 dB. Nevertheless, we compare our device performance with that of previously reported intermodal AOMs in thin-film lithium niobate [30]. At the same modulation efficiency of 18 %, the ER of our platform is evaluated to be > 24 dB which is still significantly higher than their reported ER of 10 dB.

It’s worth noting that our current devices have not yet achieved cooperative optimizations of the mode (de)multiplexer and the MRR due to the fabrication errors. Assuming the intermodal crosstalk in the mode demultiplexer and the on-resonance ER of the MRR simultaneously reach up to 20 dB by the optimization of fabrication and device design, the anticipated ER of our device could be increased to about 40 dB, approaching the level of bulk AOMs. Besides, cascading multiple MRRs at the backend of the mode demultiplexer is an optional scheme to further improve the ER based on our results. The thermal tuning efficiency in our device is measured to be only 0.34 pm/mW, unable to achieve a wide range of wavelength tuning. In future optimizations, the more efficient thermal electrode design [63] or electro-optic tuning [64] can be adopted to tune the working wavelength in MRRs efficiently.

## 5 Conclusions

In conclusion, we propose and experimentally demonstrate the high-ER mode (de)multiplexer assisted with a critical coupling MRR for the acousto-optic applications. Our designed device can be fabricated with one-step EBL and ICP etching processes. The performance of the constituent two-mode (de)multiplexer shows the on-chip IL of less than 1.25 dB and 1.92 dB for TE_0_ and TE_1_ modes at the wavelength range of 1,514–1,580 nm, respectively. The corresponding intermodal crosstalk levels for both modes are measured to be less than −20 dB. With further integrating a high-*Q* MRR at the output branch of the ADC, the measured ER of our devices can be improved over 30 dB, the maximum value reaching 35 dB with the total insertion loss of 3.25 dB. As a proof of principle, our results establish a new paradigm to practical intermodal AOMs, which advance innovative applications in on-chip nonreciprocal optical isolation [10], chip-based heterodyne detection [36], and LiDAR system [65], [66].

## Supplementary Material

Supplementary Material Details
