# Supplementary material for: High performance mode (de)multiplexer assisted with a microring resonator on the lithium niobate-on-insulator platform
Source: Nanophotonics. 2025 Jul 7;14(17):2857–67. doi: 10.1515/nanoph-2025-0146 (PMC12397742; doi:10.1515/nanoph-2025-0146)
Supplement: Supplementary file 1 — Supplementary Material Details [file j_nanoph-2025-0146_suppl_001.pdf]

# Supplementary Material: High performance mode (de)multiplexer assisted with a microring resonator on the lithium niobate-on-insulator platform

## S1. Characterization of the ADC-based mode multiplexer

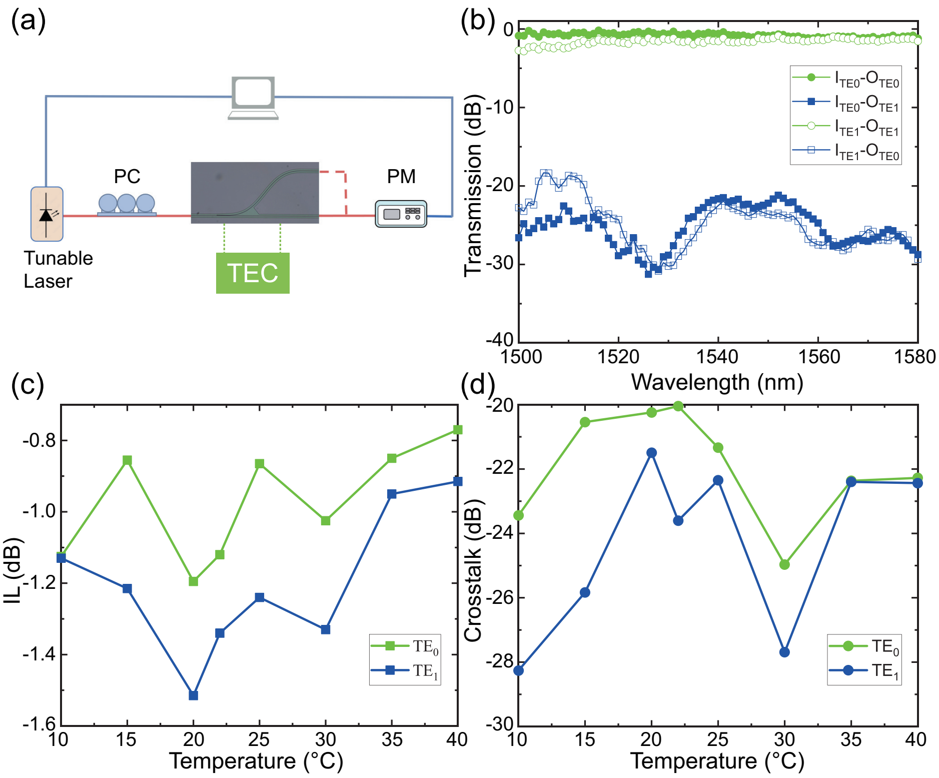


**Figure S1:** (a) Measurement setup for characterizing the device. PC: polarization controller, PM: power meter, TEC: thermoelectric controller. (b) Measured transmission spectra of the LNOI MMUX. Temperature dependence of insertion loss (c) and intermodal crosstalk (d) for $TE_0$ and $TE_1$ modes.

We characterized the performance of LNOI mode (de)multiplexers (MMUX) using the measurement setup, as depicted in Fig. S1(a). The tunable laser (Santec, TSL-775) is swept as the input light which is afterward launched into the chip via the single-mode fiber and the TE-type grating coupler. The polarization of laser is regulated with a three-paddle polarization controller (PC). The output signals are routed through the single-mode fiber and collected by an optical power meter (CommPolar, 7320B). The device temperature is managed using the thermoelectric controller (TEC) beneath

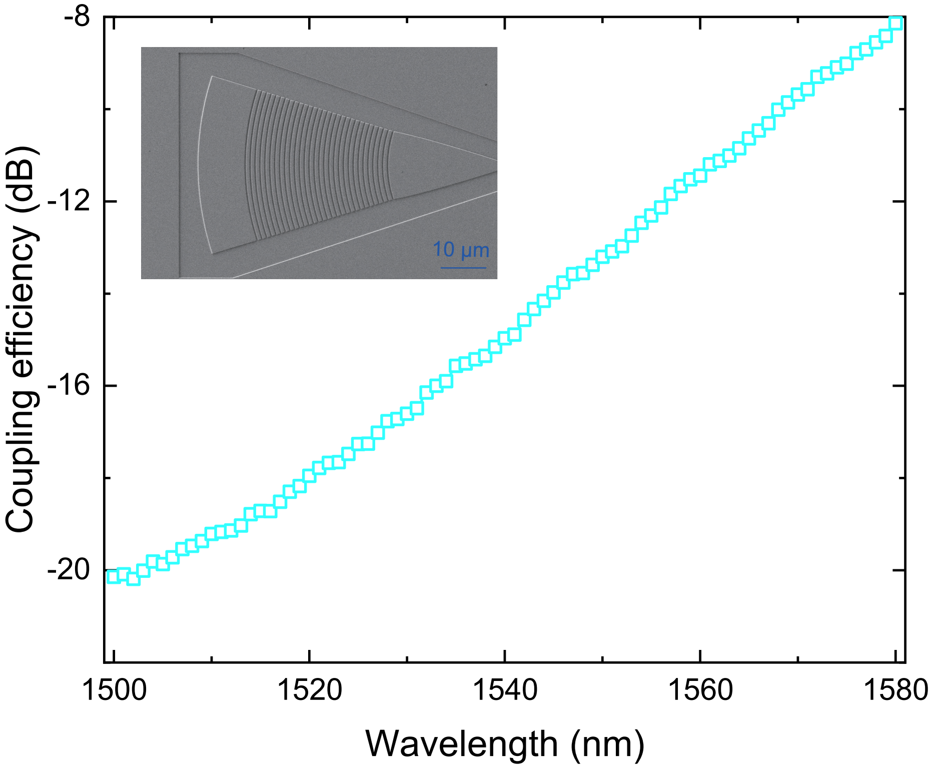


**Figure S2:** Measured coupling efficiency of the GC. The inset shows the top-view SEM image of the GC.

the sample holder. Fig. S1(b) shows the measurement results as the laser wavelength is swept from 1500 to 1580 nm. The reference waveguides with identical grating couplers (GCs) on the same chip are measured to normalize the transmission spectrum. From the measurement results, the intermodal crosstalk levels of both modes are less than -20 dB, and the on-chip insertion losses for $TE_0$ and $TE_1$ modes are less than 1.25 dB and 1.92 dB within the wavelength range of 1514-1580 nm, respectively. Specifically at 1550 nm, the crosstalk levels of $TE_0$ and $TE_1$ modes are -21.3 dB and -22.4 dB, respectively. We also measured the device performance under various temperatures from 10°C to 40°C. The temperature dependence of insertion loss and ER for $TE_0$ and $TE_1$ modes at 1550 nm is plotted in Fig. S1(c) and (d), respectively. In the whole temperature range, the insertion losses of $TE_0$ and $TE_1$ modes are less than 1.2 dB and 1.5 dB, respectively, and the measured crosstalk levels of two modes are less than -20 dB and -21.5 dB, respectively. The largest values of intermodal crosstalk can reach -28.3 dB and -23.4 dB at 10°C, the lowest temperature in our measurement setup. Therefore, our fabricated mode (de)multiplexers maintain good performance over a wide temperature range.

We employ the straight waveguide with two TE-type GCs to extract the coupling efficiency of the GC. The measured transmission spectrum of the straight waveguide includes the coupling loss of two GCs, the waveguide propagation loss, and the loss of measurement setups. Since the straight waveguide is only 950 $\mu$m long, the waveguide propagation loss is assumed to be negligible, given the extracted propagation loss from the MRR characterization. The loss of measurement setups has been carefully calibrated to obtain the coupling efficiency of the GC. Fig. S2 displays the measured coupling efficiency for one GC in the wavelength range from 1500 to 1580 nm. At 1550 nm, the fabricated GC exhibits a coupling efficiency of -13.2 dB. Although the performance of GCs is not yet fully optimized at the current stage, it does not affect the characterization of the ADC-based MMUX.

## S2. Impact of the MRR integration on insertion loss

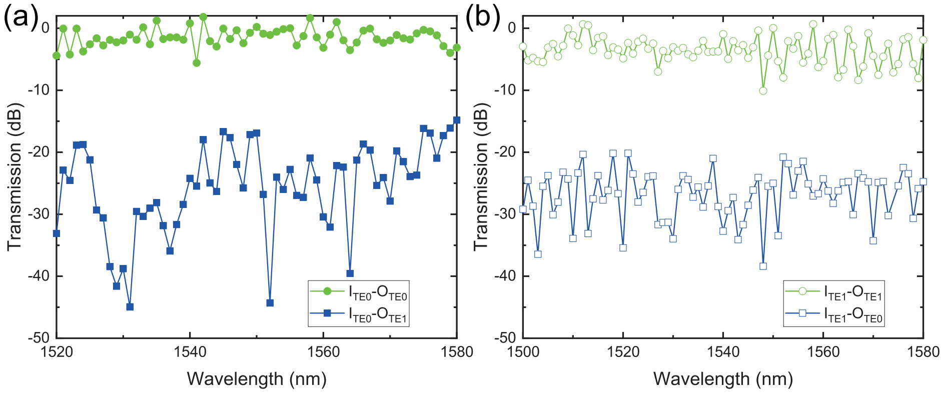


**Figure S3:** Measured transmission spectra of the ADC-based MMUX fabricated on the same chip as the integrated device. (a) Input through the $TE_0$ port. (b) Input through the $TE_1$ port.

To investigate the impact of the MRR integration on the insertion loss of the integrated device, we have compared the insertion loss performance of the ADC-based MMUX and the MMUX assisted with the MRR. Both devices are fabricated on the same chip to minimize the performance variations induced by fabrication non-uniformity. Fig. S3 displays the measured transmission spectra of the ADC-based MMUX with edge couplers. The intermodal crosstalk levels of $TE_0$ and $TE_1$ modes are less than -20 dB, consistent with the results in Fig. S1, confirming the reliability and reproducibility of our design. Table S1 summarizes the insertion loss of $TE_0$ and $TE_1$ modes for two device configurations. As shown in Table S1, the insertion loss of $TE_0$ mode remains nearly unchanged between these two devices. This is expected because the high-$Q$ MRR is integrated at the narrower access waveguide of the ADC, away from the $TE_0$ mode propagation. While for $TE_1$ mode, we observed that there is a significant increase of 1.5 dB in the average insertion loss. At the working optical wavelength (1545 nm) of the MRR, the insertion loss increases by 1.1 dB. To clarify out whether the additional loss stems from the on-chip insertion loss of the integrated MRR, we have also characterized several individual MRRs. The measured spectrum of one reference MRR is shown in Fig. S4, demonstrating the similar behavior with the high-$Q$ MRR of the integrated device. The extracted FSR is 0.743 nm, close to the measured result in Fig. 6. Additionally, the flat region of the transmission spectrum reveals the average on-chip insertion loss of about 1.4 dB denoted by the dash line, closely matching the additional loss observed in the integrated device compared with the ADC-based MMUX. Therefore, we conclude that the additional loss in the integrated device is primarily attributed to the on-chip insertion loss of the MRR.

**Table S1:** The comparison of insertion loss in the ADC-based MMUX and the MMUX assisted with the MRR.

| Insertion loss (dB) | ADC-based MMUX | MMUX assisted with the MRR |
|---|---|---|
| $TE_0$ (average) | 1.4 | 1.0 |
| $TE_0$ (1545 nm) | 0.1 | 0.8 |
| $TE_1$ (average) | 3.6 | 5.1 |
| $TE_1$ (1545 nm) | 4.8 | 5.9 |

Based on the above analyses, the insertion loss of $TE_1$ mode in the MRR-assisted MMUX ($IL_{TE1}$) comprises the on-chip insertion loss of the MRR ($IL_{MRR}$), the $TE_0$-$TE_1$ mode conversion loss of the

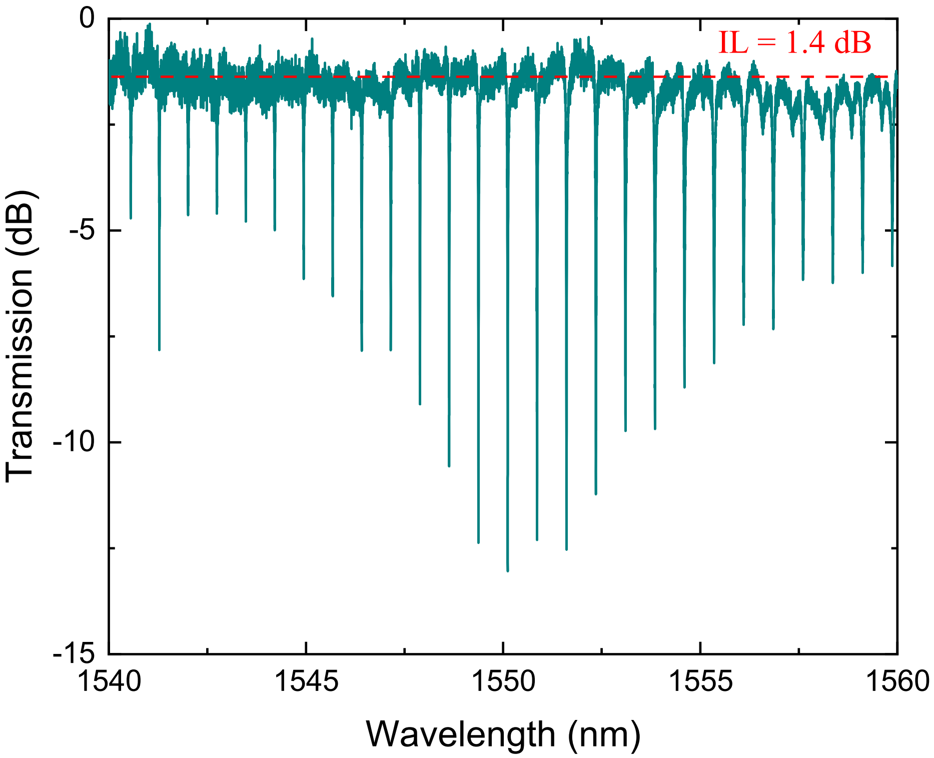


**Figure S4:** Measured spectral response of one reference MRR. The red dash line denotes the average IL.

mode multiplexer, and the succeeding $TE_1$-$TE_0$ mode conversion loss of the mode demultiplexer. Assuming the mode multiplexer behaves similarly with the mode demultiplexer in the MMUX, $IL_{TE1}$ can be expressed as:

$$IL_{TE1} = IL_{MRR} + 2 \times CL. \tag{S1}$$

Herein, CL represents the $TE_0$-$TE_1$ conversion loss in the MMUX. For the integrated device applications to AOMs, since the input signals are fed through the bus waveguide of the MMUX and $TE_0$-$TE_1$ intermodal scattering subsequently occurs in the multimode waveguide, the $TE_0$-$TE_1$ conversion of the mode multiplexer is not involved. Hence, the total insertion loss of the integrated device (IL) is given by the sum of $IL_{MRR}$ and CL:

$$IL = IL_{MRR} + CL. \tag{S2}$$

According to Eqs. (S1) and (S2), we calculated CL and the device IL to be 1.85 dB and 3.25 dB, respectively, with $IL_{MRR} = 1.4$ dB. Notably, the $TE_0$-$TE_1$ conversion loss of the integrated device is significantly higher than that of the stand-alone MMUX in Fig. S1, where the $TE_0$-$TE_1$ conversion loss is 0.62 dB at 1550 nm. To figure out this discrepancy, we performed SEM measurements on the ADC region in the MRR-assisted MMUX device, as shown in Fig. S5(a). Compared with the design parameters of the phase-matching ADC, only the access waveguide broadens by 80 nm, which will remarkably affect the mode coupling efficiency due to the breaking of phase-matching conditions, as confirmed by FDTD simulations in Fig. S5(b). Using the actual structure parameters from Fig. S5(a), the simulated mode coupling efficiency decreases to 67% (-1.74 dB), in good agreement with the experimental CL of the integrated device. Furthermore, we performed the simulations based on the actual structure parameters of the stand-alone MMUX in Fig. S1, and extracted the mode coupling efficiency to be 84% (-0.76 dB), also close to the corresponding measured value. These results suggest that the discrepancy of the $TE_0$-$TE_1$ conversion loss between two devices in Fig. S1 and Fig. 7 is indeed caused by the fabrication error.

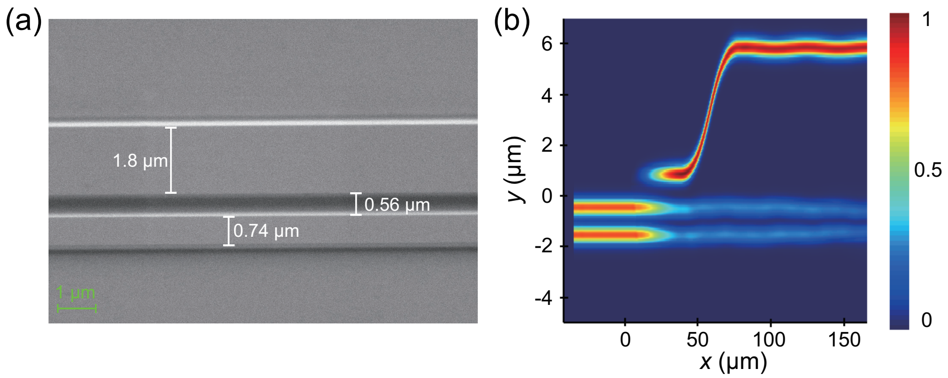


**Figure S5:** (a) SEM images of the ADC region in the MMUX assisted with the MRR. (b) The simulated light propagation at 1550 nm for $TE_1$ input, based on the simulation model using the actual structure parameters.
